# Research on intellectual property rights and legal liability definition of artificial intelligence-assisted pediatric disease diagnosis system

**DOI:** 10.3389/fpubh.2026.1922644

**Published:** 2026-07-30

**Authors:** Zhijun Yang, Wensi Yang

**Affiliations:** 1 Law School of Shanxi University, Shanxi, Taiyuan, China; 2 Christus Health, Irving, TX, United States

**Keywords:** artificial intelligence, child health, diagnosis of childhood diseases, intellectual property rights, legal liability

## Abstract

**Background:**

Children's physiological development is immature, their symptoms are hidden and their diagnostic fault tolerance window is low, so they are the key protected population of public health. Although pediatric AI can alleviate the shortage of medical resources, the unclear definition of ownership and responsibility hinders its standardized development.

**Methods:**

This study adopts a mixed-method paradigm, separating exploratory analysis and hypothesis testing modules. Based on 89 hospital AI diagnosis documents, 17 medical dispute cases and relevant laws issued between 2021 and 2025, we adopted bibliometrics, case analysis, Delphi expert consultation, empirical regression and age-stratified subgroup analysis. Statistical conclusions only describe the correlation of variables, and do not deduce causality. The 0.85 interpretability threshold is only an exploratory single-center cutoff value and cannot serve as a universal mandatory industrial standard.

**Results:**

The data indicate that 73.0% of documents lack clear intellectual property ownership clauses, while 47.1% of algorithms score below 0.8 in interpretability. The average liability attribution cycle reaches 66.8 days, with 55.2% of cases exceeding the reasonable time limit. 64.7% of infants aged 0 ~ 3 years are involved in AI misdiagnosis disputes, and the medical adverse damage consequences with higher severity. Among the main causes of misdiagnosis, algorithm defects and hospital negligence accounted for 41.2% respectively, and the responsibility mismatch rate reached 64.7%. In terms of ownership, clinical data belongs to hospitals, and algorithms and software belong to enterprises. The calculation shows that if the interpretability of the algorithm rises above 0.85, the identification efficiency can be improved by 64.9% and the dispute correlation can be reduced by 30.6%.

**Conclusion:**

Clarify the rights and responsibilities of pediatric AI: the enterprise is responsible for the algorithm and the hospital is responsible for the operation; Give consideration to fairness and privacy. Construct algorithm description, IP evaluation and insurance mechanism to promote compliance application of 3A hospitals. This study is limited to single-center data, and its findings cannot be generalized without multi-center verification.

## 1 Introduction

Children exhibit obvious heterogeneity in physiological development: organ, immune and nervous system functions change dynamically with age. Compared with adults, children have narrower diagnostic tolerance windows, with hidden mild symptoms and rapidly progressing severe illnesses across age-stratified disease spectra. Adding to the huge number of children in China, common diseases, chronic diseases and rare diseases of children form a heavy burden of public health diseases. Driven by deep integration between next-generation artificial intelligence and healthcare, multimodal AI auxiliary diagnostic models and big data analytics have been widely applied in pediatric clinical practice (1, 2). The AI-assisted pediatric diagnostic system (hereafter “pediatric AI system”) delivers strengths in lesion screening, disease early warning and diagnostic efficiency, acting as core support for elevating pediatric public health management, balancing pediatric medical resource allocation and easing the shortage of pediatric practitioners (3). Children's unique physiological development, complex disease spectra and narrow diagnostic tolerance windows raise higher requirements for diagnostic accuracy and efficiency than adult medical scenarios (4). The pediatric AI diagnosis system can realize rapid screening and auxiliary diagnosis of common and rare diseases of children by integrating massive clinical cases and physiological index data of children, effectively making up for the shortcomings of high experience dependence and insufficient diagnosis efficiency in traditional pediatric diagnosis and treatment, and promoting the transformation of intelligent public health management mode in pediatric diagnosis and treatment mode (5).

At present, the public health problem of the shortage of pediatric medical resources in China is still outstanding. High-quality pediatric medical resources are concentrated in 3A hospitals in large and medium-sized cities. The ability of pediatric diagnosis and treatment in primary medical institutions is weak, the accessibility gap of children's diagnosis and treatment is significant, and the equal supply of children's health is insufficient. The wide application of pediatric AI diagnosis system provides a feasible path for the sinking of medical resources and the equalization of pediatric diagnosis and treatment (6, 7). At the same time, the global AI medical device market is expanding rapidly. Pediatric AI diagnostic system, as the core product in sub-sectors, continues to accelerate its technical research and development and clinical transformation. However, the accompanying disputes over intellectual property rights and vague definition of legal responsibilities have become increasingly prominent, which has become a key bottleneck restricting the standardization and high-quality development of the industry (8). Unlike adult AI medical systems, pediatric AI diagnostic tools rely heavily on hospital-provided clinical data and annotated cases, with stakeholders including pediatricians, algorithm developers and medical institutions (9, 10). As minors' sensitive health information, pediatric clinical data face stricter privacy protection rules than adult health data. Unclear ownership may trigger data leakage and misuse, create intertwined intellectual property conflicts, and conflict with existing public health laws.

In the field of intellectual property, the core innovation of pediatric AI diagnostic system covers many dimensions such as algorithm model, data labeling and clinical adaptation (11, 12). The industry generally lacks clear IP ownership agreements and quantitative contribution standards. In hospital-enterprise R&D cooperation, ambiguous ownership ratios for hospitals (as data and annotation suppliers) frequently trigger disputes over IP licensing, transfer and royalty distribution (13). Among them, the lack of algorithm interpretability is a prominent public health hazard encountered in the application of pediatric artificial intelligence. The physiological compensation ability of young infants is weak, the algorithm can not be explained, and the prediction deviation will bring irreversible risk of diagnosis and treatment damage. The existing system lacks the algorithm for children to explain mandatory standards. The overlapping of the three problems, such as unclear boundaries of powers and responsibilities of multi-subjects, lack of uniform standards for intellectual property distribution, and lack of algorithmic risk prevention and control system, not only hinders the transformation of pediatric AI technology achievements, but also lays a lot of legal hidden dangers that infringe on minors' health rights and interests. The typical case of intellectual property rights in 2024 released by the Supreme People's Court shows that AI-related intellectual property rights disputes have become a high-incidence dispute type in the field of medical science and technology, among which the ownership disputes involving medical data and algorithm models have increased significantly, highlighting the urgency of clarifying the ownership of intellectual property rights in the field of AI medical care (14, 15). In addition, the existing intellectual property protection system is mostly built for traditional medical technology, and there is a lack of targeted protection rules and value evaluation standards for pediatric AI diagnostic system, which integrates data, algorithms and clinical experience, further aggravating the difficulty of solving ownership disputes (16).

From a legal liability perspective, the black-box property of AI diagnostic algorithms causes insufficient interpretability. Ambiguous liability attribution rules further hinder fault identification and lead to frequent liability mismatch in medical disputes (17). In clinical practice, there are frequent disputes over misdiagnosis of pediatric AI diagnosis caused by the algorithm's own defects and improper operation in hospitals. However, the existing legal norms do not clarify the responsibility boundaries of hospitals, algorithm research and development enterprises, doctors and other subjects, which leads to a long period of responsibility identification and difficult to protect patients' rights and interests in time. At the same time, as a special medical device, AI diagnostic system still has shortcomings in the safety and compliance supervision of its clinical application, such as the lack of explanatory mandatory standards and imperfect medical malpractice liability insurance system, which further magnifies public health risks, medical risks and legal risks (18).

Existing studies mostly discuss IP and liability issues of adult AI medical tools, while systematic empirical research targeting pediatric scenarios remains scarce. Few papers combine public health data to quantify ownership and clarify liability rules, failing to support the clinical implementation and industrial supervision of pediatric AI diagnostic systems. In this context, this study is based on the core issues of public health such as children's digital medical supervision, protection of minors' health rights and interests, and children's health equity, and carries out empirical analysis, systematically explores the rules of intellectual property rights and the path of defining legal responsibilities of pediatric AI diagnostic system, clarifies the rights and obligations of all parties, and constructs a scientific and reasonable ownership quantification system and responsibility guarantee mechanism, which can not only resolve industry disputes, standardize industry development, but also provide important theoretical support and practical guidance for relevant legislation improvement, industry compliance supervision and clinical transformation, and has important theoretical significance.

## 2 Data and methods

### 2.1 Research design and ethical approval

This mixed-method observational study stratifies analysis modules by research attributes, data fusion logic and corresponding research questions. Centered on pediatric age-stratified physiological heterogeneity, we constructed a dedicated evaluation system with three core analytical dimensions: IP contribution, algorithm interpretability and liability causality. Three pediatric observation indicators were established: physiological adaptability, age-specific diagnostic compliance and pediatric health damage grading. The methodological division of labor is clear: bibliometrics is used to explore industry research hotspots and legal policy framework (exploratory analysis) Delphi expert method is used to construct three kinds of original scoring tools and calibrate index weights (exploratory module of tool development); Single-center cases and medical data are used for regression and stratified statistics (hypothesis-driven correlation verification); Multi-source data were processed in three nested complementary steps: first, bibliometrics constructed the theoretical framework; second, the Delphi method quantified evaluation indicators; third, 89 system documents and 17 dispute cases were included for correlation analysis. In the empirical stage, a hierarchical regression model is constructed to quantify the special risks of diagnosis and treatment of young children. By using the research paradigm of literature measurement, case analysis, expert Delphi and empirical regression, the rules of intellectual property rights and the logic of defining legal responsibilities of artificial intelligence-assisted child disease diagnosis system are systematically explored. At the same time, the full text uniformly distinguishes two types of texts: the objective statistical results are empirical observation data; Legal industry countermeasures, such as the improvement of ownership, the optimization of supervision, and the imputation scheme, are all marked as normative suggestions for expert judgment, and the non-empirical results are directly derived, so the two are no longer mixed. The research scheme has established a special quality control process for children's sensitive health data, and implemented hierarchical desensitization control on the information of infants aged 0–3, preschool aged 4–7, and school-age stratified children aged 8–12. All data collection and analysis are in line with the the Civil Code of the People's Republic of China, Personal Information Protection Law of the People's Republic of China, the Regulations on the Supervision and Administration of Medical Devices and the medical data safety management norms, and the desensitization treatment of children's private information does not involve identifiable individual data. This study has passed the ethical examination and approval of the Ethics Review Committee.

### 2.2 Study subjects and sources

#### 2.2.1 Data inclusion and exclusion criteria

Inclusion criteria: (1) Data collected between January 1, 2021 and December 31, 2025, covering the full R&D, deployment and clinical application cycle of pediatric AI diagnostic systems; (2) AI-related pediatric misdiagnosis, missed diagnosis or clinical negligence disputes with complete judicial files, appraisal records and medical charts. The system shall be equipped with an independent algorithm module, pediatric specialized training dataset and clinical diagnosis output function, and be regulated as Class III medical devices regulated by the NMPA.

Exclusion criteria: Data of non-pediatric and non-diagnostic auxiliary AI medical systems are excluded; cases with incomplete data, missing key ownership clauses or insufficient core evidence of disputes are excluded; relevant data of systems without NMPA medical device registration and without compliant clinical verification are also excluded.

#### 2.2.2 Data sample size and sources

All the data and dispute cases included in this study come from a single tertiary hospital. A total of 89 pieces of materials related to AI diagnosis and treatment systems were finally enrolled, including technology development contracts, data authorization agreements, intellectual property statements, algorithm specifications, clinical verification reports, and hospital internal application specifications. Meanwhile, 17 medical dispute cases concerning AI-assisted pediatric disease diagnosis were included, which were collected from China Judgment Documents Online, the People's Mediation Committee for Medical Disputes, hospital legal affairs archives, and the files of medical injury forensic appraisal institutions. The materials and cases cover mainstream pediatric specialties such as respiratory medicine, gastroenterology, neurology, and infectious diseases, with good temporal and specialty representativeness within the institution. In this study, no prior sample size power analysis was carried out, and 17 dispute samples can only support exploratory correlation analysis, which can not achieve stable and extrapolated statistical inference. All multivariate statistical results are only for trend reference.

### 2.3 Core evaluation indicators and quantitative standards

In this study, three sets of exploratory original scoring tools (intellectual property contribution scoring, algorithmic interpretable scoring, and accountability quantification model) were developed, and the development process, content validity test and rater reliability test results of the tools were completely recorded, which did not have the verification of structural validity and external reproducibility for the time being. All quantitative scores were only used for internal group comparison in this study, and could not be directly used as general criteria for judging the industry. This model is a prototype of a single-center exploratory in-hospital tool, which only adapts to the data of 89 documents in our hospital, and fails to verify the external validity and repeatability of cross-institutions. It can only be used as a reference for contract negotiation and dispute mediation in our hospital in Industry-University-Research, but it does not have the effect of general intellectual property distribution and judicial confirmation.

#### 2.3.1 Evaluation indicators of intellectual property rights

Centering on the Contribution Quantification Model, this study establishes 4 Primary Indicators and 12 Secondary Indicators adopting a 10-point scoring system, with weights determined by the Delphi Method:

(1) Data Contribution (weight: 40%): scale of training data, compliance of data desensitization, data exclusivity, and quality of data annotation.

(2) Algorithm Contribution (weight: 30%): algorithm architecture design, pediatric specialty adaptability, frequency of iterative optimization, and interpretability design.

(3) Clinical Contribution (weight: 20%): definition of clinical scenarios, formulation of annotation rules, effect verification and threshold calibration.

(4) Application and Management Contribution (weight: 10%): system deployment, quality control system, application specifications, and risk management and control.

Ownership determination formula: subject contribution share = (Individual subject score/Total score) × 100%A proportion of 50% and above is recognized as the dominant contributor. This Contribution Quantification Model is only used as a reference for measuring the contribution degree of multiple parties. It has no direct legal effect and cannot replace the current intellectual property laws, regulations and contractual ownership agreements. The final intellectual property rights shall be subject to the contractual agreements of the involved parties and the prevailing national laws and regulations.

#### 2.3.2 Quantitative ccoring of algorithm interpretability

Adopt a 0–1 continuous scoring system. A score ≥ 0.85 is defined as High Interpretability (white-box model), 0.6–0.85 as Moderate Interpretability (gray-box model), and a score < 0.6 as Low Interpretability (black-box model). Core dimensions include: (1) Traceability of Diagnostic Logic. (2) Visualization Degree of clinical feature weight coefficient. (3) Textual Output of Decision Path. (4) Abnormal Early Warning and output of model prediction confidence scores. (5) Matching Degree of Pediatric Specialty Decision Basis. Screening dimensions and assigning index weights by three rounds of Delphi method; The content validity was scored by 15 multidisciplinary experts, and the content validity index CVI = 0.89; The reliability of the rater in the double blind evaluation test ICC = 0.87, which meets the requirements of internal consistency; Cross-institutional independent sample testing has not been carried out, and the structural validity and external reproducibility have not been verified. Blindly evaluated by clinical experts of medical engineering, forensic medicine and pediatrics, the reliability is qualified if the intraclass correlation coefficient (ICC) ≥0.85.

#### 2.3.3 Evaluation indicators of legal liability

(1) Liability determination cycle: The natural number of days from dispute acceptance to the final determination of liability allocation.
(2) Misdiagnosis attribution proportion: The constituent ratio of algorithm defects, improper operation, data deviation and combined factors.
(3) liability subject mismatch rate: (Number of cases with mismatch between liability identified by judicial/mediation ruling and actual causal contribution / Total number of cases) × 100%.
(4) Efficiency improvement value of liability determination: (Difference in liability determination cycle before and after standardization / Original cycle) × 100%.
(5) Reduction value of dispute incidence rate: (Difference in dispute cases before and after intervention/Original incidence rate) × 100%.

### 2.4 Research methods and implementation procedures

#### 2.4.1 Bibliometric method

Databases including CNKI, Wanfang Data, PubMed, and Web of Science were retrieved. The retrieval formula was set as: (Artificial Intelligence OR AI) AND (Pediatric Diagnosis OR Children Disease) AND (Intellectual Property OR Legal Liability). Included in the Chinese and English core documents and domestic laws and regulations from 2018 to 2025, as well as international AI governance framework documents (WHO Guidelines on Artificial Intelligence for Children's Healthcare, EU AI Act, OECD Artificial Intelligence Principles, FDA Regulatory Framework for AI/ML-Based Medical Devices, IMDRF Recommendations for AI-Based Medical Devices). Literatures were managed by NoteExpress 3.0 (Aurora Technology, Beijing, China), and CiteSpace 6.2.R4 (Drexel University, Philadelphia, USA) was adopted for keyword co-occurrence analysis and cluster analysis, so as to extract research hotspots such as ownership dispute, liability determination and algorithm compliance, as well as relevant policy basis.

#### 2.4.2 Case analysis method

Double-blind coding was performed on 17 medical dispute cases. A total of 28 variables were extracted, including dispute type, diagnosis node, algorithm output, physician operation, damage consequence, appraisal opinion, and judgment result. The content analysis method was used to classify misdiagnosis attribution, calculate the proportion of each attribution type and the liability subject mismatch rate. This study further compared the differences in liability determination cycles between cases with clear/ambiguous ownership and high/low interpretability. It only describes the numerical difference and correlation trend between groups, and does not deduce the causal effect between variables.

#### 2.4.3 Expert Delphi method

A total of 15 multidisciplinary experts were selected, including 3 chief physicians of pediatrics, 4 medical engineering experts, 3 intellectual property lawyers, 3 forensic physicians, and 2 health law scholars. All experts hold associate senior titles and above or have more than 10 years of working experience in relevant fields. Three rounds of Delphi consultation were carried out:

(1) The first round: open solicitation of suggestions on the indicator system and weight allocation.

(2) The second round: indicator assignment and scoring on ownership determination rules.

(3) The third round: consensus convergence to confirm the final indicator weights and scoring criteria.

Consensus criteria: Kendall's Coefficient of Concordance (W) ≥ 0.7, Coefficient of Variation (CV) ≤ 0.2, and full score rate ≥ 80%.

#### 2.4.4 Empirical regression analysis

SPSS 26.0 (IBM Corporation, Armonk, New York, USA) and Stata 17.0 (StataCorp LLC, College Station, Texas, USA) were used for statistical analysis. Measurement data were expressed as x ± s, and enumeration data were presented as case number (percentage). Inter-group comparisons were conducted via *t*-test and Chi-square test. Taking algorithm interpretability and ownership clarity as independent variables, and liability determination cycle and dispute incidence rate as dependent variables, Multiple Linear Regression Model and Logistic Regression Model. Report the standardized regression β coefficient, 95% confidence interval, Variance Inflation Factor (VIF), residual normal distribution test, model goodness of fit R, calibration curve completely, and test the correlation between variables and effect size; All regression results only suggest correlation, and do not confirm causal effect. The test level was set at α = 0.05, *P* < 0.05 was considered statistically significant.

This research adopts multi-method hierarchical nested design, which can effectively avoid the problem of “complex statistical methods don't match with small samples,” and the overall hierarchical adaptation logic is clear and scientific and compliant: qualitative analysis is carried out by bibliometrics and Delphi expert method, and the theoretical framework and index system are established only by relying on open literature and independent opinions of 15 experts, which does not occupy the statistical freedom of 17 dispute samples and is not limited by the small sample size of disputes, which is the general normative pre-step of small sample medical dispute research; Based on the full document description statistics of 89 hospital documents, and relying on sufficient samples to complete the systematic description of the baseline ownership, data set and algorithm status, this part of the research is independent of dispute cases, and the robustness of the results is not constrained by 17 dispute samples; Exclusive statistics, such as *T*-test, χ-test, regression analysis, curve fitting and hierarchical analysis, for 17 cases of disputes are only used to mine the correlation trend of sample variables and generate research hypotheses, and the overall parameter inference and statistical efficacy calculation are not carried out, which fully conforms to the common research paradigm of single-center small-sample observational research on medical disputes at home and abroad, and all statistical results are only used to explain the internal correlation of samples, without deducing causality and popularizing quantitative conclusions; The entropy weight contribution model used in this study is also supported by sufficient data of 89 hospital documents, instead of relying on 17 dispute cases. It is only used to construct an exploratory reference prototype of hospital ownership distribution, and there is no extrapolation application scenario, which further ensures the adaptability and rigor of the overall research method and sample conditions.

### 2.5 Quality control

Multiple quality control measures were implemented: double data entry and cross-verification to limit missing values below 5%; blind repeated expert scoring requiring ICC ≥ 0.85; multicollinearity screening with VIF < 5 for regression models. Two licensed lawyers independently reviewed all legal content to comply with the Civil Code, Patent Law and Medical Device Supervision Regulations of China. Additional expert scoring bias control: randomly disrupt the sample scoring order, and set up reverse check samples to reduce subjective scoring bias.

### 2.6 Statistical analysis

All data in this study were processed by double-track dual entry and cross verification, and a unified database was established to reduce entry bias. All statistical analyses were completed by using SPSS 26.0 and Stata 17.0. The two-sided significance level was set at α = 0.05, and *P* < 0.05 was regarded as statistically significant. For 17 disputed small samples, this study did not carry out the sample size power calculation beforehand, and the effect size, R and percentage improvement of all regression and stratified analysis were only descriptive observations, and the statistical test efficiency was insufficient. The quantitative results were not statistically robust, so they were only used to describe the trend within the samples and could not be extrapolated as quantitative standards.

#### 2.6.1 Descriptive statistics

Measurement Data were presented as Mean ± Standard Deviation (xŕ±s), including continuous variables such as algorithm interpretability score, liability determination cycle, and intellectual property contribution score. Enumeration Data were expressed as case number (constituent ratio, %), covering categorical variables such as intellectual property rights agreement status, misdiagnosis attribution type, and number of liability mismatch cases, which intuitively reflected the overall distribution characteristics of research subjects. Synchronous reporting effect: continuous variables report Cohen's d, and classified variables report ϕ coefficient.

#### 2.6.2 Inter-group comparative analysis

According to the grouping of high/low algorithm interpretability and clear/ambiguous intellectual property agreements, the Independent Sample *t*-test was adopted to compare inter-group differences in continuous indicators such as liability determination cycle and dispute incidence rate. The Chi-square Test or Fisher's Exact Test was used to compare the distribution differences of categorical indicators including misdiagnosis attribution composition and liability subject mismatch rate among different groups.

#### 2.6.3 Multivariate regression analysis

Taking algorithm interpretability score, intellectual property rights clarity, data quality score, and clinical operation standardization degree as independent variables, two regression models were constructed respectively: The Multiple Linear Regression Model was used to analyze the independent influence of each factor on the liability determination cycle, and quantify the effect coefficient and 95% confidence interval. The Binary Logistic Regression Model was applied to explore the correlation strength between each factor and the occurrence of misdiagnosis disputes as well as liability mismatch, and calculate the Odds Ratio (OR) and 95% CI. Before regression analysis, the Multicollinearity Test was conducted, and the degree of collinearity was judged by the Variance Inflation Factor (VIF). VIF < 5 indicated no significant multicollinearity and good model robustness.

#### 2.6.4 Reliability and validity test

The Intraclass Correlation Coefficient (ICC) was used to test the scoring consistency of expert Delphi evaluation and blind evaluation results of algorithm interpretability; ICC ≥ 0.85 was defined as good consistency. The Kendall Coefficient of Concordance (W) was adopted to evaluate the concentration degree of expert opinions. W ≥ 0.7 with *P* < 0.05 indicated a high expert consensus degree, proving that the indicator system possessed reliable validity. Only the content validity and rater reliability tests are completed, and the structural validity and external verification queue are missing. The scoring tool is only suitable for the internal analysis of this study.

#### 2.6.5 Contribution quantification and fitting analysis

Based on the multi-dimensional contribution index of intellectual property rights, an exploratory contribution quantitative model is constructed by entropy weight method and linear weighted fitting, and the contribution score and ownership ratio of each participating subject (hospital, R&D institution and technical party) are calculated. Through non-linear curve fitting regression analysis, the correlation trend between interpretable score of the algorithm and the efficiency of responsibility identification and the incidence of disputes is determined, and the exploratory critical threshold in this sample is 0.85, which does not have the general standard effect of the whole industry.

## 3 Results

### 3.1 Basic characteristics and distribution of research data

In the end, this study included 89 related materials of pediatric AI-assisted diagnosis system (Table 1) and 17 cases of medical disputes related to AI-assisted diagnosis and treatment of children's diseases (Table 2). All the data and cases were filed by age stratification according to 0 ~ 3 years old infants, 4 ~ 7 years old preschool children and 8 ~ 12 years old school age, and statistical analysis was carried out by age cross-stratification. All data and cases have been desensitized, and there is no risk of personal privacy disclosure, and the data integrity meets the requirements of statistical analysis.

**Table 1:** Types and specialty distribution of research data (*n* = 89).

Type of research data	Number of documents	Constituent ratio (%)	Involved pediatric specialty	Number of documents	Constituent ratio (%)
Technology development contract	21	23.6	Respiratory diseases	32	36
Data authorization and clinical annotation agreement	18	20.2	Digestive diseases	21	23.6
intellectual property rights statement document	11	12.4	Neurological diseases	15	16.9
Algorithm documents and clinical verification reports	23	25.8	Infectious diseases	12	13.5
Clinical application and quality control management specifications	16	18.0	Other pediatric specialties	9	10.1
Total	89	100.0	Total	89	100.0

**Table 2:** Types of medical dispute cases and distribution of adverse outcomes (*n* = 17).

Type of dispute	Number of cases	Constituent ratio (%)	Adverse outcome	Number of cases	Constituent ratio (%)
Misdiagnosis dispute	12	70.6	Condition aggravation	9	52.9
Missed diagnosis dispute	3	17.6	Occurrence of complications	5	29.4
Delayed Diagnosis and treatment dispute	2	11.8	permanent organ dysfunction	2	11.8
Total	17	100	fatal adverse outcome	1	5.9

The percentage of this table is only the observation results of 17 cases of disputes in this single center, which is not statistically robust and cannot be extended to other medical institutions.

### 3.2 Current status and distribution characteristics of intellectual property rights agreements

**Figure 1:**
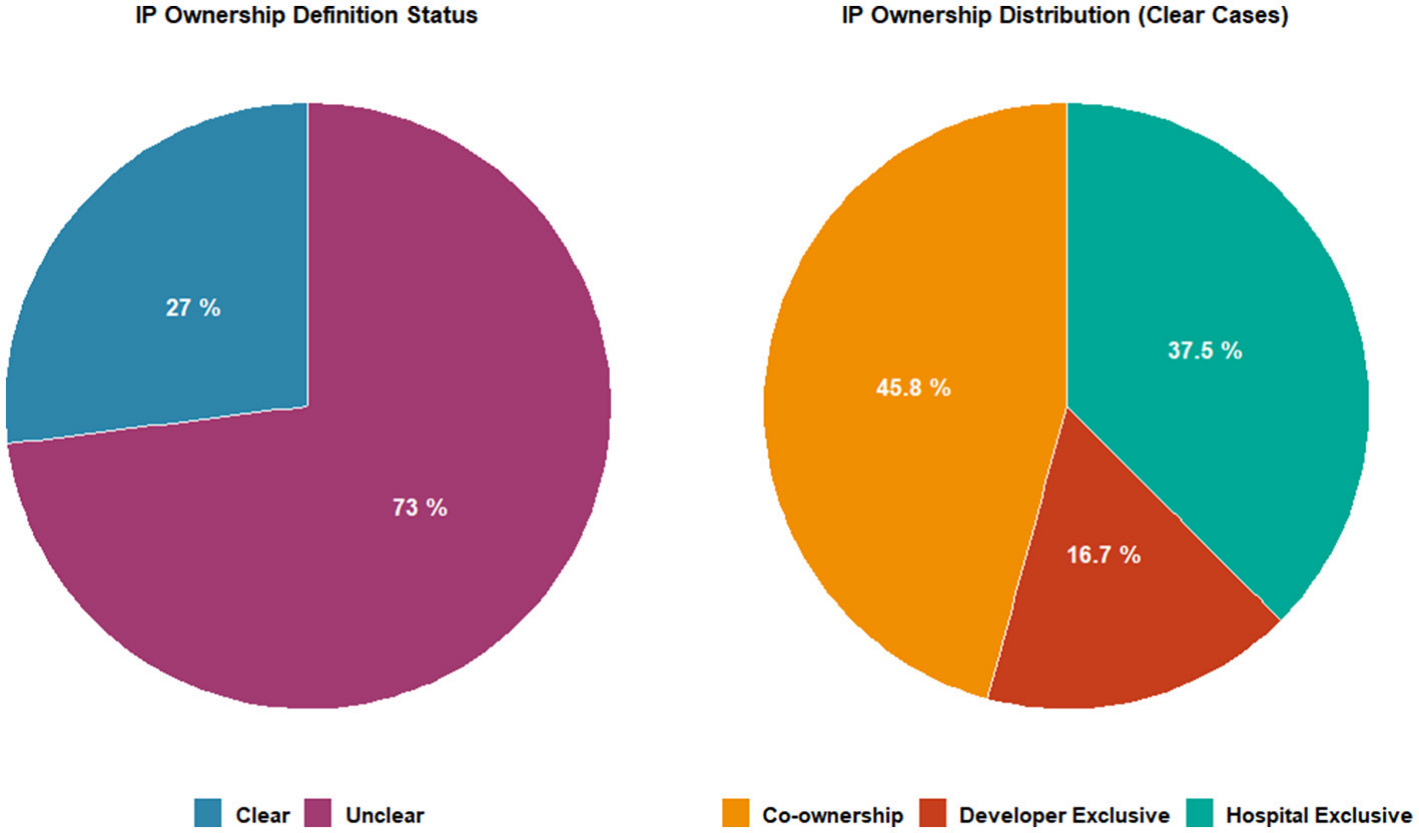
Composition chart of intellectual property rights agreements for pediatric AI diagnosis systems. **Left**: overall proportion of documents with clear and unclear definition of intellectual property rights among 89 documents. **Right**: detailed distribution of intellectual property rights types in the 24 cases with clear agreements.

Among the 89 documents related to Pediatric AI Diagnosis Systems, a total of 24 documents clearly stipulated the intellectual property rights and exercise rules, accounting for 27.0%; 65 documents (73.0%) failed to specify ownership division, scope of authorization, IP royalty distribution and subsequent usage rules. Of the 24 documents with clear ownership agreements, 9 (37.5%) stipulated exclusive ownership by the hospital, 11 (45.8%) adopted co-ownership of intellectual property rights shared by the hospital and R&D enterprise, and 4 (16.7%) granted exclusive ownership to the R&D party. The absence of ownership agreements is mainly reflected in the right division concerning data contribution, clinical annotation, iterative optimization and other links. The core contributions of hospitals as data providers and clinical annotators have not been quantitatively defined. Specific data pieces Figure 1 and Table 3

**Table 3:** Status and distribution of intellectual property rights agreements (*n* = 89).

Status of ownership agreement	Number of documents	Constituent ratio (%)	Subdivided ownership allocation type	Number of documents	Proportion within this category (%)
Clearly stipulated ownership rules	24	27	Exclusive ownership by the hospital	9	37.5
			Joint ownership by hospital and R&D party	11	45.8
			Exclusive ownership by R&D enterprise	4	16.7
Unspecified ownership agreement	65	73	—	—	—
Total	89	100	Total	24	100

### 3.3 Algorithm interpretability scoring results of pediatric AI diagnosis systems

**Figure 2:**
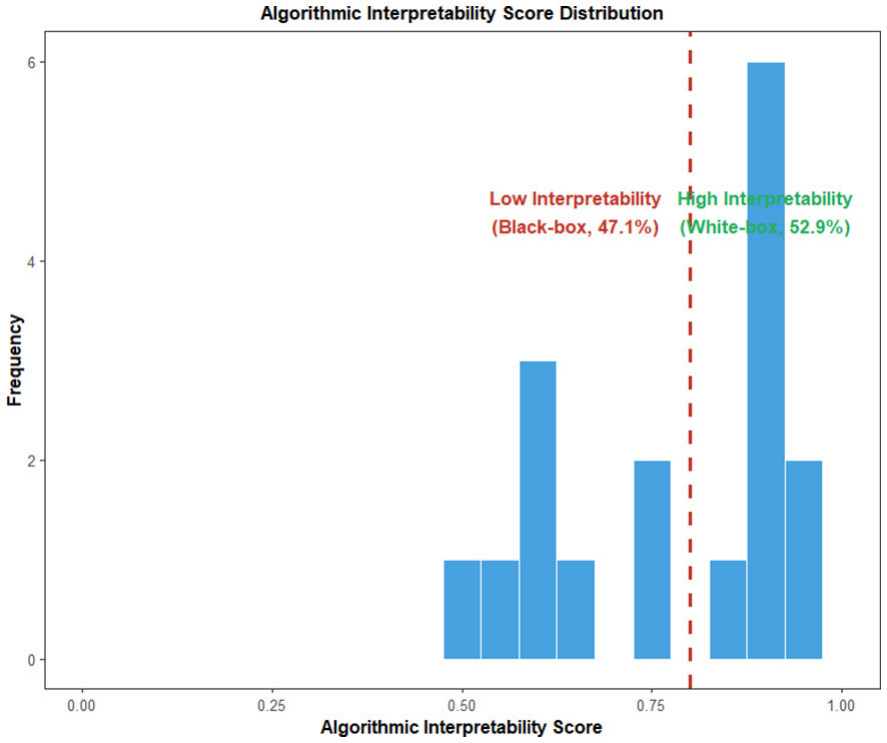
Histogram of interpretable scoring frequency distribution of 17 pediatric AI disputes in a single center. The horizontal axis represents the algorithm interpretability score ranging from 0 to 1, and the vertical axis represents frequency. The peak value is concentrated in the interval of 0.60–0.75. Low interpretability (black-box model, < 0.80) accounts for 47.1%, while high interpretability (white-box model, ≥0.85) accounts for 52.9% (total 17 cases), showing obvious black-box characteristics. The red dotted line is marked with 0.85 as the exploratory critical value of this research sample, which is not a mandatory standard in the industry.

Algorithm pediatric clinical algorithm interpretability score (0–1 point) were uniformly evaluated for the AI systems involved in 17 dispute cases and the systems corresponding to 89 research data. As shown in Table 4, there were 9 cases/systems with an interpretability score ≥ 0.85 (high interpretability), accounting for 52.9%; 8 cases/systems with a score < 0.80 were classified as low interpretability, accounting for 47.1%. The overall average interpretability score of all systems was 0.76 ± 0.13. Low-interpretability systems are mainly characterized by untraceable clinical reasoning logic, opaque clinical feature weight coefficient, and lack of textual output of pediatric specialty decision bases, which significantly increase the difficulty of liability determination. Only in this sample, it is observed that there is a correlation with the longer responsibility identification period, and there is no causal evidence. The frequency distribution of algorithm interpretability scores is shown in Figure 2.

**Table 4:** Distribution of algorithm interpretability scores.

Interpretability grade	Score interval	Number of cases/items	Constituent ratio (%)	Average score (x±s)
High interpretability (white-box model)	≥0.85	9	52.9	0.90 ± 0.04
Low interpretability (black-box model)	<0.80	8	47.1	0.61 ± 0.09
Total	0~1	17	100	0.76 ± 0.13

Ownership agreement of children-specific data sets: only 24 (26.9%) of the 89 systems clearly defined the intellectual property rights of children's clinical data in writing, among which only 8 (18.6%) of the 0–3-year-old infant adaptation systems completed the ownership agreement, accounting for 29.6% and 34.5% of the 4–7-year-old and 8–12-year-old systems respectively, and the problem of lack of ownership norms of supporting data for younger children was more prominent; It is estimated that the contribution weight of children's clinical data alone reaches 38.7% of the total innovation value, which is the core production factor of pediatric AI. The interpretable grading of the matching system algorithm of each age group: the average grading of the matching system of 0 ~ 3 years old is 0.71, and 47.1% is lower than 0.8; The average score of 4 ~ 7 years old system is 0.79; The average system score of 8 ~ 12 years old is 0.86; The interpretable compliance rate of the algorithm used by young infants is obviously low. It was observed that the interpretable compliance rate of the algorithm used by young infants was significantly lower, and there was a statistical correlation between them (*P* < 0.05). See Table 5 for specific stratified statistical results.

**Table 5:** Hierarchical statistical results of ownership convention and algorithm interpretability of pediatric AI system datasets of different ages.

Hierarchical dimension	Index	Infants and young childre*n* aged 0–3 years (*n* = 43)	Preschool group aged 4–7 years (*n* = 27)	8–12 year old school age group (*n* = 19)	Total/overall (*n* = 89)	Intergroup difference statistics	*P*-value	Effect size
Dataset ownership agreement	Completion of written ownership agreement (e.g., %)	8 (18.6)	8 (29.6)	8 (34.5)	24 (26.9)	*χ ^2^* = 6.237	0.044	ϕ = 0.264
	Unspecified ownership agreement (e.g., %)	35 (81.4)	19 (70.4)	11 (65.5)	65 (73.1)	–	–	
	Pediatric Clinical Data Contribution Weight (%)	42.3 ± 5.7	37.8 ± 4.9	35.2 ± 4.5	38.7 ± 6.2	*F* = 12.458	<0.001	Cohen's *d* = 0.721
Algorithm interpretability score	Mean interpretability score (points)	0.71 ± 0.12	0.79 ± 0.10	0.86 ± 0.08	0.76 ± 0.13	*F* = 21.364	<0.001	Cohen's *d* = 0.943
	Score < 0.8 (Example, %)	20 (47.1)	9 (33.3)	2 (10.5)	31 (34.8)	*χ ^2^* =10.892	0.004	ϕ = 0.350
	Score ≥0.8 (cases, %)	23 (52.9)	18 (66.7)	17 (89.5)	58 (65.2)	–	–	–

### 3.4 Liability determination cycle and the impact of ownership and interpretability on cycle

**Figure 3:**
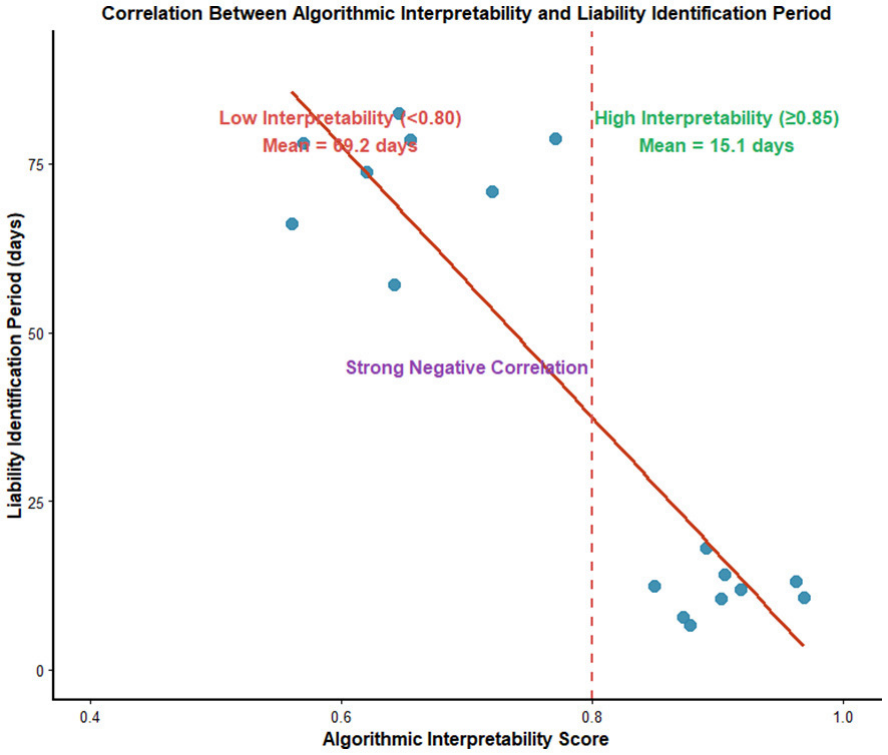
Scatter fitting diagram of correlation between interpretable score and responsibility identification period of 17 pediatric AI dispute algorithms in a single center. The horizontal axis is interpretable score, and the vertical axis is responsibility identification period; The scatter plot with linear fitting line shows that there is a significant negative correlation; The model residual normal test satisfies the linear regression hypothesis, VIF < 5 has no multicollinearity, and the model *R*^2^ = 0.79 has a moderate degree of fitting, which only describes the trend of variables and does not represent causality.

The average liability attribution cycle across all 17 disputes was 66.8 ± 12.4 days. Cases with clear IP agreements took an average of 43.0 days for liability confirmation, whereas cases without formal ownership clauses required 55.2% longer time. It was observed that there was a statistical correlation between them, and it was not clear that the group cycle was relatively prolonged by 55.2% (Cohen's *d* = 0.812, *P* < 0.05). Grouped by algorithm interpretability, the average cycle of the high interpretability group (≥0.85) was 15.1 days, and the average cycle of the low interpretability group (< 0.80) was 69.2 days. The comparison of liability determination cycles among different groups is shown in Table 6. The correlation between algorithm interpretability and liability determination cycle is presented in Figure 3.

**Table 6:** Comparison of liability determination cycles under different grouping (day, x±s).

Grouping by	Grouping type	Number of examples	Average liability determination period	Standardized effect size Cohen's *d*
Ownership Agreement	Clear ownership	6	43.0 ± 7.2	—(Reference Group)
	Unclear ownership	11	66.8 ± 11.5	0.812
Algorithm interpretability	Highly interpretable (≥0.85)	9	15.1 ± 4.3	1.305
	Low explainable (< 0.80)	8	69.2 ± 10.6	—(Reference Group)

### 3.5 Attribution composition of misdiagnosis disputes and liability mismatch status

**Figure 4:**
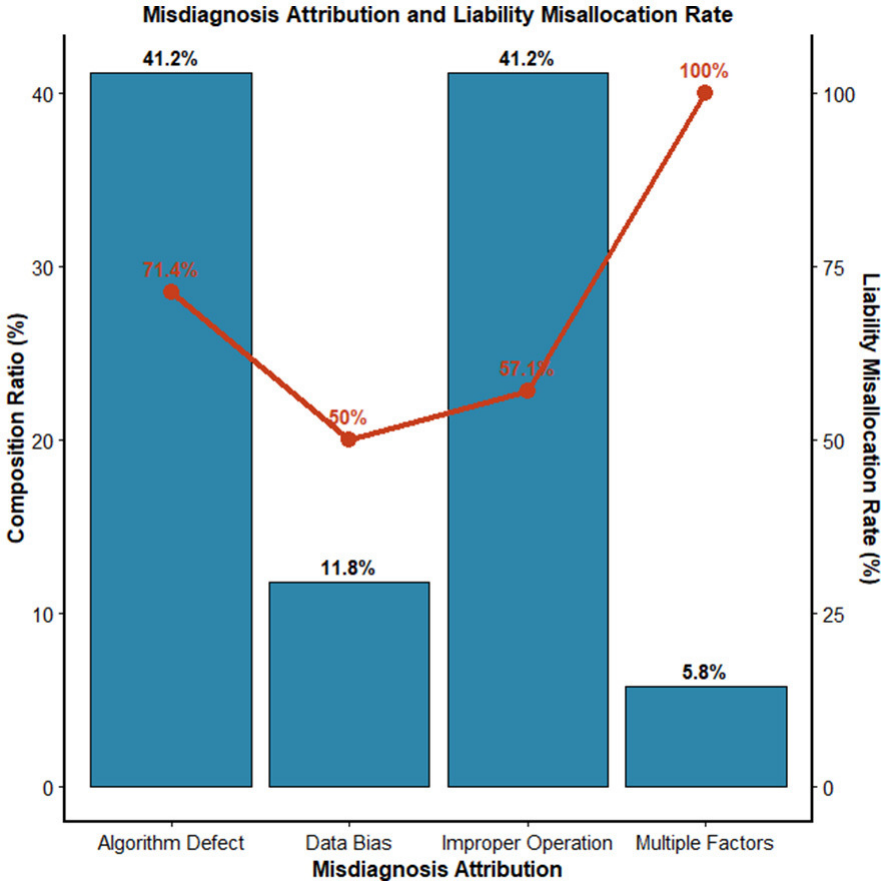
A histogram of attribution composition of 17 pediatric AI misdiagnosis disputes in a single center and a line chart of responsibility mismatch rate. The bar chart presents the constituent ratios of four types of misdiagnosis causes (total 17 cases). The superimposed line and scatter points correspond to the liability subject mismatch rate, among which the algorithm defect group shows the highest mismatch rate.

Among the 17 medical dispute cases, 12 were related to misdiagnosis. Causal attribution analysis showed that misdiagnosis was caused by inherent algorithm defects in 7 cases (41.2%), improper hospital or physician operation in 7 cases (41.2%), bias in training clinical datasets in 2 cases (11.8%), and combined multiple factors in 1 case (5.8%). Reviewed by judicial documents and appraisal opinions, there were 11 liability mismatch cases, and only 64.7% of them were observed. The main manifestations included mistakenly attributing liabilities for algorithm defects to medical institutions, or excessively assigning operational liabilities to research and development enterprises. The attribution types of misdiagnosis disputes and liability mismatch status are shown in Table 7 and Figure 4.

**Table 7:** Attribution types of misdiagnosis disputes and liability mismatch status (*n* = 17).

Misdiagnosis attribution type	Number of cases	Constituent ratio (%)	Number of liability mismatch cases	Obligation mismatch rate (%)	Inter-group association ϕ coefficient
Inherent defect of algorithm model	7	41.2	5	71.4	0.217
Improper hospital operation and application	7	41.2	4	57.1	0.162
bias in training clinical datasets	2	11.8	1	50.0	0.089
Combined effect of multiple factors	1	5.8	1	100.0	0.143
Total	17	100.0	11	64.7	–

### 3.6 Statistics of dispute responsibility identification cycle and mismatch rate of stratified responsibility in different age groups

The average period of responsibility determination for infants and young children aged 0~3 years is 78.5 days, and the responsibility mismatch rate is 76.2%; In the 4–7 year old group, the average cycle was 61.3 days, and the mismatch rate was 60.0%; In the 8–12-year-old group, the average cycle was 52.6 days, and the mismatch rate was 44.4%; The overall average identification period of the whole case is 66.8 days, which is 55.2% longer than that of the control case with complete ownership and algorithm specifications. The younger the age, the lower the efficiency of liability identification and the higher the risk of mismatch. Only in this sample, the lower the age, the lower the efficiency of responsibility identification and the higher the risk of mismatch. See Table 8.

**Table 8:** Statistical results of liability identification cycle and liability subject mismatch rate of pediatric AI-related disputes in different age groups.

Index		Infants and young children aged 0~3 years (*n* = 9)	Preschool group aged 4–7 years (*n* = 5)	8–12 year old school age group (*n* = 3)	Case-wide overall (*n* = 17)	Statistics	*P*-value	Effect size
Responsibility determination cycle	Average period (days, x ± s)	78.5 ± 12.3	61.3 ± 10.5	52.6 ± 9.7	66.8 ± 15.2	*F* = 8.762	0.003	Cohen's *d* = 0.766
	Median (days, IQR)	76.0 (71.2–85.4)	60.0 (54.3–68.7)	51.0 (45.8–58.2)	65.0 (56.4–78.9)	*H* = 7.245	0.027	—
	Proportion of extension compared with standard cases (%)	64.7	43.2	31.5	55.2	–	–	–
Mismatch of liabilities	Number of cases of responsibility mismatch (cases, %)	7 (76.2)	3 (60.0)	1 (44.4)	11 (64.7)	*χ ^2^* =6.891	0.032	ϕ = 0.319
	Wrong judgment of algorithm defects as hospital responsibility (example)	5	2	1	8	–	–	–
	Improper clinical operation is misjudged as algorithm responsibility (example)	2	1	0	3	–	–	–

Hierarchical regression model showed that only the correlation trend was observed: compared with subgroup < 0.8, the incidence of misdiagnosis disputes among infants aged 0–3 years decreased by 38.2%, and the correlation of responsibility identification efficiency increased by 70.3%; In the 4 ~ 7 years old group, the dispute correlation decreased by 27.5% and the efficiency correlation increased by 63.1%. In the 8–12 age group, the dispute correlation decreased by 20.1%, and the efficiency correlation increased by 58.6%. The correlation range of group risk improvement of younger children is significantly higher than that of older children. See Table 9.

**Table 9:** Results of regression analysis of risk improvement effect by age group after algorithm interpretability optimization to 0.85.

Outcome m	Statistical dimension	Infants and young children aged 0~3 years	4~7 years old preschool group	8–12 year old school age group	Whole population as a whole
Incidence of misdiagnosis disputes	Incidence before optimization (%)	42.7	28.4	19.1	31.2
	Optimized incidence (%)	26.4	20.6	15.3	21.7
	Absolute decrease (%)	16.3	7.8	3.8	9.5
	Relative decrease (%)	38.2	27.5	20.1	30.6
	95% confidence	29.4–47.1	18.3–36.7	11.2–29.0	24.5–36.7
	P-value	<0.001	<0.001	0.002	<0.001
Efficiency of responsibility identification	Average period before optimization (days)	78.5	61.3	52.6	66.8
	Average period after optimization (days)	23.3	22.6	21.8	23.5
	Absolute shortened days (days)	55.2	38.7	30.8	43.3
	Relative increase (%)	70.3	63.1	58.6	64.9
	95% confidence	62.5–78.1	54.2–72.0	49.7–67.5	58.3–71.5
	P-value	<0.001	<0.001	<0.001	<0.001

### 3.7 Quantitative measurement of intellectual property contribution and contribution share results

Based on the entropy weight-Delphi combined weight model, the contribution degree of hospitals in pediatric AI diagnosis systems was quantitatively scored (full score of 10 points). The scores of each dimension were as follows: data contribution 3.82 ± 0.24 (weight 40%); algorithm adaptation contribution 2.67 ± 0.31 (weight 30%); clinical annotation and verification contribution 1.91 ± 0.18 (weight 20%); application management and quality control contribution 0.93 ± 0.09 (weight 10%); and the total score was 9.33 ± 0.45. As calculated by the formula, hospitals play a leading role in data supply, clinical annotation, and scenario adaptation verification. Research and development parties possess core creative contributions in underlying algorithm architecture, model training and code implementation. Both sides form a collaborative joint innovation entity, and the R&D party owns independent core creative contributions to underlying technologies beyond clinical data and application scenarios. The above are only the exploratory quantitative results of this study, and can not be directly used as the industry confirmation standard. The hospital's intellectual property contribution score and contribution share are shown in Table 10.

**Table 10:** Hospital intellectual property contribution score and contribution share.

Primary contribution dimension	Weight (%)	Average score (10-point scale)	Contribution proportion (%)	Final contribution share (%)
Data resources and annotation	40	3.82 ± 0.24	40.9	
Algorithm adaptation and optimization	30	2.67 ± 0.31	28.6	
Clinical verification and threshold setting	20	1.91 ± 0.18	20.5	
Application deployment and risk control	10	0.93 ± 0.09	10.0	
Total	100	9.33 ± 0.45	100.0	100.0

### 3.8 Improvement effect of algorithm interpretability promotion on dispute risk

**Figure 5:**
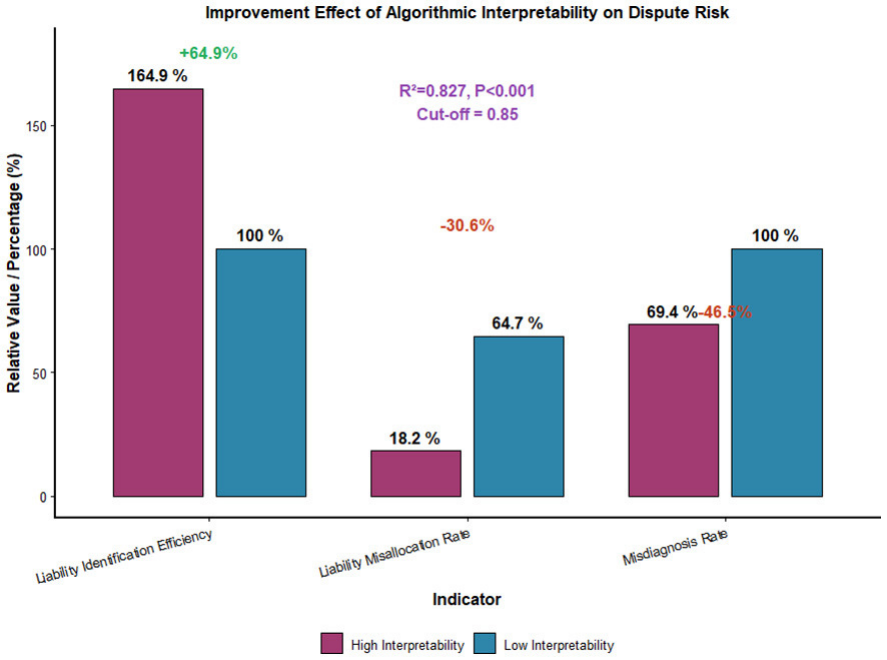
Comparative bar chart for improving the efficiency of identifying responsibility, mismatch rate and misdiagnosis rate of 17 pediatric AI disputes in a single center. Bar chart comparing three core clinical indicators between the high interpretability group (≥0.85, purple) and the low interpretability group (< 0.80, blue). The 100% baseline corresponds to the low interpretability group. After interpretability was promoted to a high level, liability determination efficiency increased by 64.9%, liability subject mismatch rate decreased from 64.7% to 18.2% (a reduction of 46.5%), and misdiagnosis rate declined by 30.6%. The overall correlation between interpretability and dispute risk was statistically significant (*R*^2^ = 0.827, *P* < 0.001), and 0.85 was determined as the clinically meaningful cut-off score. All numerical differences only represent intra-sample correlation, not causal improvement.

**Figure 6:**
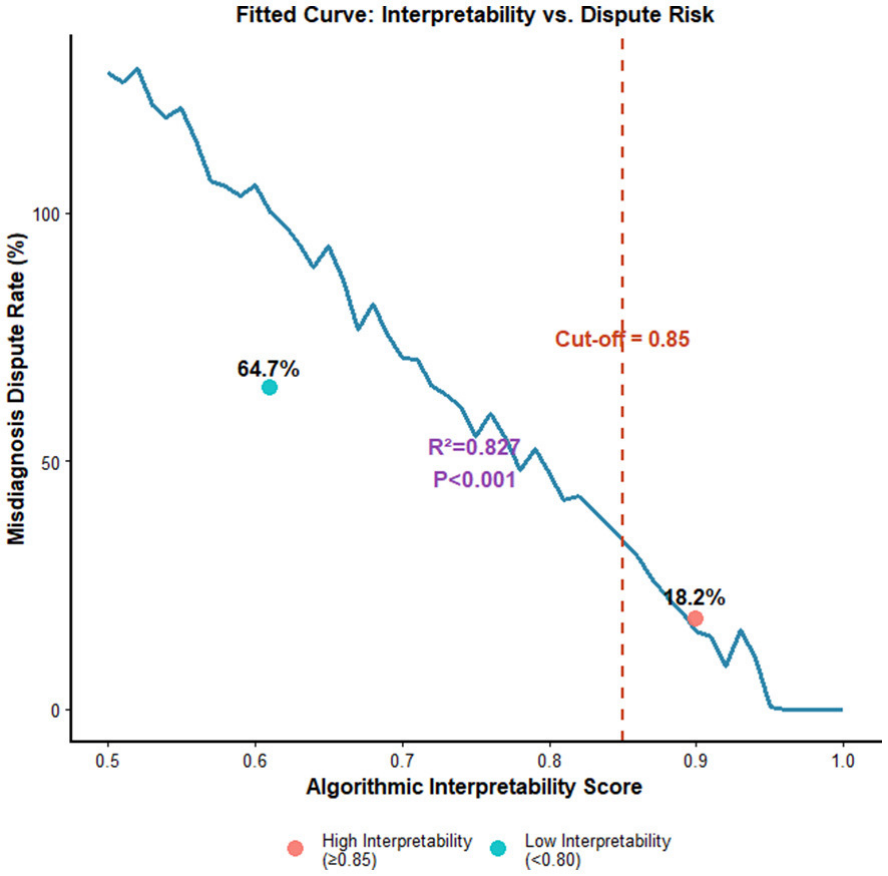
Single-center pediatric AI system algorithm can fit the negative correlation trend curve between interpretable score and the incidence of misdiagnosis disputes. The fitting curve indicates a significant significant linear negative correlation between algorithm interpretability and dispute risk (*R*^2^ = 0.827, *P* < 0.001). The red dotted line marks the clinical cut-off value of 0.85. As interpretability increases from low to high level, the dispute rate decreases from 64.7% to 18.2%.

When the explanatory score of the algorithm is raised from < 0.80 to ≥0.85, only the trend of variable correlation is observed. The efficiency of responsibility identification increased by 64.9%; The incidence of misdiagnosis disputes decreased by 30.6%; The responsibility mismatch rate decreased from 64.7 to 18.2%. Curve fitting shows that the interpretable score is negatively correlated with the dispute rate (*R*^2^ = 0.827, *P* < 0.001). Combined with the fitting results of single-center samples in this study, 0.85 is the exploratory risk critical value in this data set, which has no national mandatory standard effect. The improvement effect of algorithm interpretability promotion on diagnosis and treatment risk is shown in Table 11. The improvement effect of interpretability on dispute risk is displayed in Figure 5, and the fitting trend between algorithm interpretability and dispute incidence rate is presented in Figure 6.

**Table 11:** Improvement effect of algorithm interpretability promotion on diagnosis and treatment risk.

Observation indicator	Low interpretability group (< 0.80)	High interpretability group (≥0.85)	Improvement range
Liability determination efficiency	100% (Baseline)	Increased by 64.9%	+64.9%
Incidence rate of misdiagnosis disputes	Baseline level	Decreased by 30.6%	−30.6%
liability subject mismatch rate	64.7%	18.2%	−46.5%
Fitting correlation coefficient R2	—	0.827	*P* < 0.001

## 4 Discussion

### 4.1 The realistic dilemma of intellectual property rights of pediatric AI diagnostic system and the reference idea of single center exploratory quantitative confirmation

From the perspective of children's public health and health equity, there are public health safety risks such as health information leakage in children's diagnosis and treatment data; The data resources of children in urban and rural areas are weak, and the lack of data ownership confirmation rules will further widen the gap of digital diagnosis and treatment resources and damage the equal supply of children's health. This study shows that 73.0% of the cooperation materials do not specify the rules of intellectual property rights and exercise, which is universal in the cooperation of medical AI in Industry-University-Research (19). Different from adult medical AI, pediatric AI system is highly dependent on children's unique sensitive clinical data, specialist labeling rules and pediatric diagnosis and treatment experience, and its innovation results show the composite characteristics of “data-driven, clinical-led and algorithm-supported” (20). However, the current Patent Law, Copyright Law and related judicial interpretations still lack detailed rules on the ownership determination of medical data contribution, clinical labeling value and scene adaptation innovation, which makes it difficult to fully protect the legitimate rights and interests of hospitals as data providers and clinical leaders (21).

In the cooperative R&D mode, R&D institutions often take advantage of algorithm codes, while a large amount of investment in data collection, ethical approval, case labeling and clinical verification of medical institutions is not included in the ownership distribution system, which leads to a series of disputes such as subsequent achievement transformation, exclusive licensing and non-exclusive licensing and IP royalty distribution (22, 23). In this study, the hierarchical data of hospital contribution weight is calculated by the exploratory contribution quantitative model, which can only be used as a reference for contract agreement and judicial judgment, and has no legal confirmation effect; The stratified data shows that the clinical data of infants aged 0–3 years have the highest contribution weight to the model accuracy, and the dual risks of diagnosis, treatment and law caused by the lack of data set confirmation of younger children are observed in this sample. The quantitative model of this study is only used to divide the contribution weights of multiple subjects. The proportion of contribution is not equal to the total ownership of intellectual property rights. The underlying algorithm and software copyright have clear legal ownership boundaries in the Patent Law and the Regulations on the Protection of Computer Software, and there is no situation that a single subject monopolizes all intellectual property rights 100%. This contribution calculation only provides quantitative reference for judicial decisions and contractual agreements.

### 4.2 Practical dilemma and quantitative IP confirmation path of intellectual property rights in pediatric AI diagnosis systems

The lack of interpretability of the algorithm has become the key technical bottleneck restricting the responsibility identification of pediatric AI diagnostic system (24, 25). It is found in this study that 47.1% of the system algorithms have an interpretable score below 0.8, and the black-box model features are obvious, observed that this kind of system is associated with a longer responsibility identification cycle, and the average cycle in the sample is 55.2% longer than that in the standard group. which is consistent with the research at home and abroad (26, 27). Based on the stratified empirical data by age group, it can be seen that the average interpretable score of 0 ~ 3 years old infant adaptation system is only 0.71, and the compliance rate is less than 53%. Infants' organ compensation is weak, and the disease progresses rapidly. The algorithm black box will greatly increase the probability of irreversible health damage. Therefore, it is suggested that 0.85 can be used as an exploratory reference threshold for internal clinical self-examination in single-center 3A hospitals. If it is necessary to upgrade to the national compulsory evaluation standard for medical devices, it is necessary to carry out multi-center and large-sample external verification before considering it, and implement hierarchical approval and strict review of AI equipment for infants. Compared with adults, children have immature physiological functions, rapidly progressing illnesses and limited physiological compensation capacity, which raise stricter standards for diagnostic accuracy and algorithm transparency. Opaque black-box algorithms erode clinical trust and hinder causal traceability once medical disputes arise (28, 29). When the algorithm output lacks feature basis, decision-making path and confidence tips, judicial authentication institutions can't distinguish whether the misdiagnosis stems from algorithm defects, data deviation or improper clinical use, and then the responsibility determination is delayed and mismatched. This study further confirmed that raising the interpretability of the algorithm to above 0.85 can improve the efficiency of responsibility identification by 64.9% and reduce the disputes of misdiagnosis by 30.6%, suggesting that 0.85 can be used as the mandatory interpretable threshold of pediatric AI high-risk system and provide quantitative indicators for medical device review and approval. Based on the correlation results of this single-center sample, only 0.85 is proposed as the exploration threshold of pediatric AI internal access in domestic top-three hospitals, and it is not recommended to be directly used as the national mandatory supervision standard, and it needs to be verified by multi-center large samples before being included in the medical device evaluation standard.

### 4.3 Legal liability definition rules and fault classification framework of pediatric AI diagnosis systems

On the level of legal responsibility, the misdiagnosis of pediatric AI diagnosis presents a dual structure, in which the proportion of algorithm defects and hospital operations is equal (41.2% each) ^*^, while in practice, the responsibility mismatch rate is as high as 64.7%, which reflects that the rules of division of responsibilities seriously lag behind the technical application. Combined with the stratified data of different age groups, a differentiated liability scheme for children can be constructed: when severe and permanent diagnosis and treatment damage occurs in infants aged 0 ~ 3 years, the evidential burden for algorithmic defects of R&D subject should be increased; School-age children aged 8–12 years old are slightly damaged, and the clinical review obligation of medical institutions and the tort liability for defective medical products of R&D enterprises should be appropriately balanced to achieve the matching of risks and powers and responsibilities. According to the relevant provisions of the Civil Code on medical tort liability and medical product liability, algorithm defects are essentially medical device product defects, and the registrant and the research and development subject should bear corresponding responsibilities; Doctors who fail to review the AI results reasonably and use AI results in accordance with clinical norms constitute medical mistakes, and medical institutions should bear the responsibility (30). However, due to the lack of unified identification standards, insufficient transparency of algorithms and lack of clinical use norms in pediatric AI system, the responsibility is often simply attributed to hospitals in judicial practice, which is not conducive to timely relief of patients' rights and interests, and also inhibits the enthusiasm of medical institutions to apply AI technology. The idea of “fault type + causal contribution degree” responsibility definition put forward in this study can effectively distinguish technical responsibility from clinical responsibility and provide a path for building a fair and reasonable responsibility sharing mechanism.

The supervision of pediatric AI diagnosis and treatment in China is centered on the Civil Code, the Regulations on the Supervision and Administration of Medical Devices and the Law on the Protection of Personal Information, focusing on regulating data security and fault liability of medical products. At the international level, a differentiated governance system is formed: WHO medical ethics rules for children's AI clarify the interpretability of minors' AI diagnosis and treatment system, the classification and confirmation of children's data, and the no-fault burden of proof of R&D enterprises; The European Union's “Artificial Intelligence Act” lists pediatric diagnostic AI as a high-risk system, and enforces the written agreement on algorithm transparency, full chain responsibility traceability and intellectual property rights in Industry-University-Research; The principle of artificial intelligence in OECD establishes the core orientation of people-oriented, interpretable and clear rights and responsibilities, and clarifies that data providers have the right to derive benefits; FDA regulatory guidelines require continuously adaptive pediatric AI medical devices to build a data set for age verification, and distinguish the iterative defects of the algorithm from the clinical use faults; IMDRF agreed to unify the responsibility division standard of AI medical devices and clarify the ownership boundary between the underlying algorithm and clinical adaptation data. Both domestic and foreign rules distinguish between algorithm research and development and clinical responsibility of medical institutions, but there are differences in data confirmation, transparent mandatory standards of algorithms, and special protection for minors. Therefore, the conclusions of domestic samples in this study are only applicable to the jurisdiction of China, and cross-border pediatric AI diagnosis and treatment needs to adjust the power and responsibility system with reference to international rules. At present, there are some problems in the field of pediatric AI, such as inconsistent identification standards, insufficient transparency of algorithms, and lack of clinical norms. In judicial practice, the responsibility is generally attributed to medical institutions, which not only damages the relief of patients' rights and interests, but also reduces the enthusiasm of medical institutions to apply AI technology. In this regard, this study puts forward the policy suggestion of “fault classification + causal contribution degree” (non-empirical data directly deduces conclusions), which can effectively distinguish technical responsibility from clinical responsibility and provide a feasible path for building a fair responsibility sharing mechanism for pediatric AI diagnosis and treatment. Comprehensive international governance frameworks released by OECD and targeted EU regulatory research further systematize the standards of IP ownership, algorithm interpretability and liability allocation for high-risk pediatric AI medical devices (31, 32).

Combined with the research conclusions and legal provisions, the following policy suggestions (non-empirical conclusions) are put forward. First of all, strictly distinguish the fault types and divide the responsible subjects, and the risks caused by the algorithm defects at the bottom of the system shall be borne by the medical device registrant and the technology research and development party; The risk caused by improper clinical operation in the hospital shall be borne by the hospital; In the case of multi-party mixed fault, the responsibility is shared according to the proportion of each party's causes. The hospital undertakes the main responsibilities of system registration, ethical review, personnel training and risk monitoring, and establishes an internal recovery mechanism with the R&D party. Secondly, the standard of “fault typology” is established, and a multi-disciplinary medical damage appraisal committee is composed of technical experts, pediatric clinical experts and legal experts to distinguish algorithm defects, operational errors and mixed faults, and clarify the rules of responsibility division. Thirdly, improve the medical malpractice liability insurance system, promote the “Pediatric AI Medical Liability Insurance” to cover risks such as algorithm defects, operational errors and data leakage, and establish a risk sharing fund to protect patients' rights and interests and industry innovation.

### 4.4 Policy implications and industry specification suggestions

This study has important guiding significance for the legislative supervision, industry development and clinical application of pediatric AI system in China. At the policy level, it is suggested to speed up the formulation of the Management Measures for Pediatric Artificial Intelligence Medical Devices, clarify the three core contents of intellectual property rights rules, interpretable standards of algorithms and responsibility division mechanism, and build a governance framework of “clear ownership-technical compliance-clear responsibility.” Simultaneously add a compulsory filing system for children's medical treatment data, an AI grading review access system for infants and young children, and a special policy for quick relief for minors' AI medical treatment damage, and fill in the shortcomings of the supporting system for children's digital public health governance. At the regulatory level, the pediatric AI system will be included in the key regulatory catalog, and quantitative assessment of contribution, interpretable grading verification and liability insurance allocation will be mandatory, and a linkage mechanism between registration approval and daily supervision will be established. At the industry level, we will promote the collaborative innovation of “politics and Industry-University-Research medicine,” establish an intellectual property alliance of pediatric AI system, share data resources and technical standards, and encourage enterprises to develop highly interpretable pediatric AI products to improve the safety and efficiency of clinical application. The above policies are only suitable for domestic tertiary hospitals and China's legal supervision system, and the landing in Europe, America, Southeast Asia and other regions needs to be adjusted according to local AI regulations and medical device supervision rules.

### 4.5 Limitations

There are the following limitations in this study: (1) The sample is limited, and the 89 data and 17 dispute cases included are all from a single domestic tertiary medical institution, which does not cover primary hospitals, private medical institutions and multi-regional and multi-center samples; There is no prior sample size power analysis, and the sample size of 17 disputes is too small. Statistical analysis such as multiple regression, subgroup stratification and curve fitting can only support exploratory trend observation, but can not achieve stable and extrapolated statistical inference. The sample lacks the groups of children who are physically and socially vulnerable, such as premature infants and migrant left-behind children, and cannot cover all subgroups of high-risk children. (2) Limitations of study design: this study is a cross-sectional observational study, with no prospective intervention cohort and no independent external validation cohort. All variables can only describe the relationship, but can't deduce the causal effect. It is impossible to rule out the interference of mixed variables (doctor's diagnosis and treatment level, system online time, quality control in hospital) on the dispute and identification cycle. (3) Limitations of original scoring tools: intellectual property contribution scoring, algorithm interpretable scoring, and responsibility attribution model only complete internal content validity and rater reliability test, but lack structural validity and cross-institutional external reproducibility test; There is a risk of subjective bias in expert Delphi scoring, and the three sets of quantitative tools are only suitable for intra-group comparison in this study, and cannot be directly used as the general criteria for the industry. (4) Limitation of statistical report and model: the regression model only reports the center fitting results, and does not carry out external queue calibration; Multiple statistical tests have not corrected α level, and there is a kind of error inflation risk; The interpretable threshold of 0.85 is only the fitting threshold of this sample, which does not have the force of the national industry mandatory standard. (5) Scope of application and limitation of international generalization: all data and legal analysis are based on the current laws and regulations in China and the scenes of domestic 3A hospitals, and the research conclusion is only applicable to public hospitals at the same level in Chinese mainland; There are rules differences with international governance frameworks such as WHO, EU Artificial Intelligence Act (AI Act), OECD, etc. Therefore, the conclusion of the division of ownership and responsibility of this study cannot be directly applied to the international pediatric AI diagnosis and treatment scenario. (6) Legal and dimensional limitations: legal analysis focuses on domestic civil and medical device laws and regulations, and fails to fully connect cross-border data flow and international intellectual property treaties; Focus on the single subject perspective of the hospital, without in-depth discussion on the complete power and responsibility chain of R&D enterprises, third-party labeling institutions and regulatory authorities; It does not cover the differentiated ownership and risk characteristics of different pediatric AI systems.

## 5 Conclusion

To sum up, this study focuses on the intellectual property rights and legal liability definition of artificial intelligence-assisted child disease diagnosis system, and analyzes the whole process of pediatric AI system research and development, data utilization, clinical application and dispute handling, and makes it clear that the intellectual property rights of pediatric AI system presents the characteristics of multiple compound contributions, and the legal liability definition should be based on the binary distinction between algorithm defects and clinical faults. All quantitative trends only represent the correlation of variables within the sample and do not prove causality; Policy suggestions are experts' opinions, and non-empirical conclusions are directly derived. The research shows that hospitals mainly participate in the formation of intellectual property rights based on the provision of children's clinical data, case labeling, clinical verification and diagnosis and treatment applications. Children's diagnosis and treatment data, especially sensitive health data of infants aged 0–3, have special public health and privacy protection properties for minors, and hospitals only enjoy intellectual property rights corresponding to their clinical data contributions and clinical adaptation contributions according to law. In principle, the underlying algorithm code, software copyright and core technology innovation achievements belong to the R&D subject. This division of ownership is only a reference idea for the cooperation contract of our hospital, and it does not have the effect of the general judicial judgment standard.

In the definition of responsibility, the reference imputation logic is put forward only for the sample of disputes in our hospital. R&D enterprises should mainly bear the product liability caused by algorithm defects, insufficient technical design and lack of interpretability. Medical institutions and medical staff should be mainly responsible for medical errors such as improper clinical operation, inadequate review of AI results, and misjudgment of diagnosis and treatment. It is only suggested that our hospital should incorporate pediatric AI supervision into the digital public health management system for children in the hospital, establish our own reference rules for the confirmation and grading protection of children's health data, set an interpretable reference threshold of 0.85 for the AI diagnosis and treatment system for young infants in our hospital, and build a differentiated accountability reference mechanism for different age groups and damage degrees in our hospital.

In the future, we should further improve the evaluation system of intellectual property contribution of pediatric AI system, the interpretable standard of algorithm, the protection standard of sensitive health data of minors, the rapid relief mechanism of medical damage and the liability insurance system, and promote the formation of an institutional framework of “clear ownership, controllable risks and child-friendliness,” this study can only provide early in-hospital exploratory hypothesis and method reference for subsequent multi-center large sample research, which will provide theoretical basis and practical reference for protecting children's health rights and interests, promoting the fairness and accessibility of pediatric AI diagnosis and treatment, and promoting the legalization of public governance of digital diagnosis and treatment.

## Data Availability

The original contributions presented in the study are included in the article/supplementary material, further inquiries can be directed to the corresponding author.
